# The unusual first sign of presentation of hepatocellular carcinoma: a rare case report

**DOI:** 10.3389/fonc.2026.1907582

**Published:** 2026-08-26

**Authors:** Rocco Morra, Anna Nappi, Nicoletta Zanaletti, Mauro Piccirillo, Luca Tarotto, Salvatore Stilo, Francesco Fiore, Francesco Izzo, Antonio Avallone

**Affiliations:** 1 Clinical Experimental Abdominal Oncology Unit, Istituto Nazionale Tumori IRCCS Fondazione G. Pascale, Naples, Italy; 2 Division of Hepatobiliary Surgery, Istituto Nazionale Tumori IRCCS Fondazione G. Pascale, Naples, Italy; 3 Division of Interventional Radiology, Istituto Nazionale Tumori IRCCS Fondazione G. Pascale, Naples, Italy

**Keywords:** case report, hepatocellular carcinoma, multidisciplinary approach, sphenoid sinus metastasis, undiagnosed cancer

## Abstract

**Background:**

Hepatocellular carcinoma (HCC) is the most common primary liver malignancy, characterized by aggressive biological behavior and a high propensity for extrahepatic spread. The most common sites of metastasis are the lungs, regional lymph nodes, bones, adrenal glands, and peritoneum. In contrast, the paranasal sinuses are an unusual site for metastatic HCC, and involvement of the sphenoid sinus represents an even rarer event.

**Case description:**

We report the case of a 67-year-old male patient who presented with transient diplopia, left eyelid ptosis, and refractory epistaxis as the initial clinical manifestations of a previously undetected HCC. Histopathological evaluation of the sinus mass and subsequent imaging confirmed a metastatic lesion from a silent primary liver tumor. The patient was managed using a multidisciplinary approach, undergoing radiation therapy to the sphenoid sinus for pain control and endovascular embolization of the sphenopalatine arteries for refractory epistaxis. In March 2026, a contrast-enhanced computed tomography (CT) scan revealed new osteolytic lesions, consistent with bone metastases, involving the proximal third of the left clavicle and the T9 vertebral body. The patient subsequently received further palliative radiotherapy to the left clavicle and T7-T9, and initiated first-line systemic therapy with atezolizumab plus bevacizumab, completing four cycles. Due to disease progression at multiple skeletal sites and in the sphenoid region, as revealed by a CT scan in June 2026, second-line treatment with lenvatinib was initiated and is ongoing at the latest follow-up.

**Conclusions:**

Despite its rarity, the differential diagnosis of a paranasal sinus lesion should include the possibility of metastasis from HCC, particularly in patients with underlying chronic liver disease. Histopathological confirmation and a multidisciplinary approach are critical for optimizing therapeutic planning in this challenging clinical scenario.

## Introduction

Hepatocellular carcinoma (HCC) is the sixth most common cancer globally and the third leading cause of cancer-related death worldwide (1). Over 90% of HCC cases arise in patients with underlying chronic liver disease. The main risk factors include chronic hepatitis B or C viral infections, alcohol-related liver disease, and metabolic dysfunction-associated fatty liver disease. The clinical presentation of HCC is often insidious and nonspecific, frequently including abdominal pain, general malaise, anorexia, weight loss, nausea, vomiting, jaundice, ascites, hepatomegaly, and splenomegaly (2). Because of its insidious onset and inadequate surveillance in high-risk patients, such as those with metabolic etiology, HCC is often diagnosed at an advanced stage.

**Figure 1:**
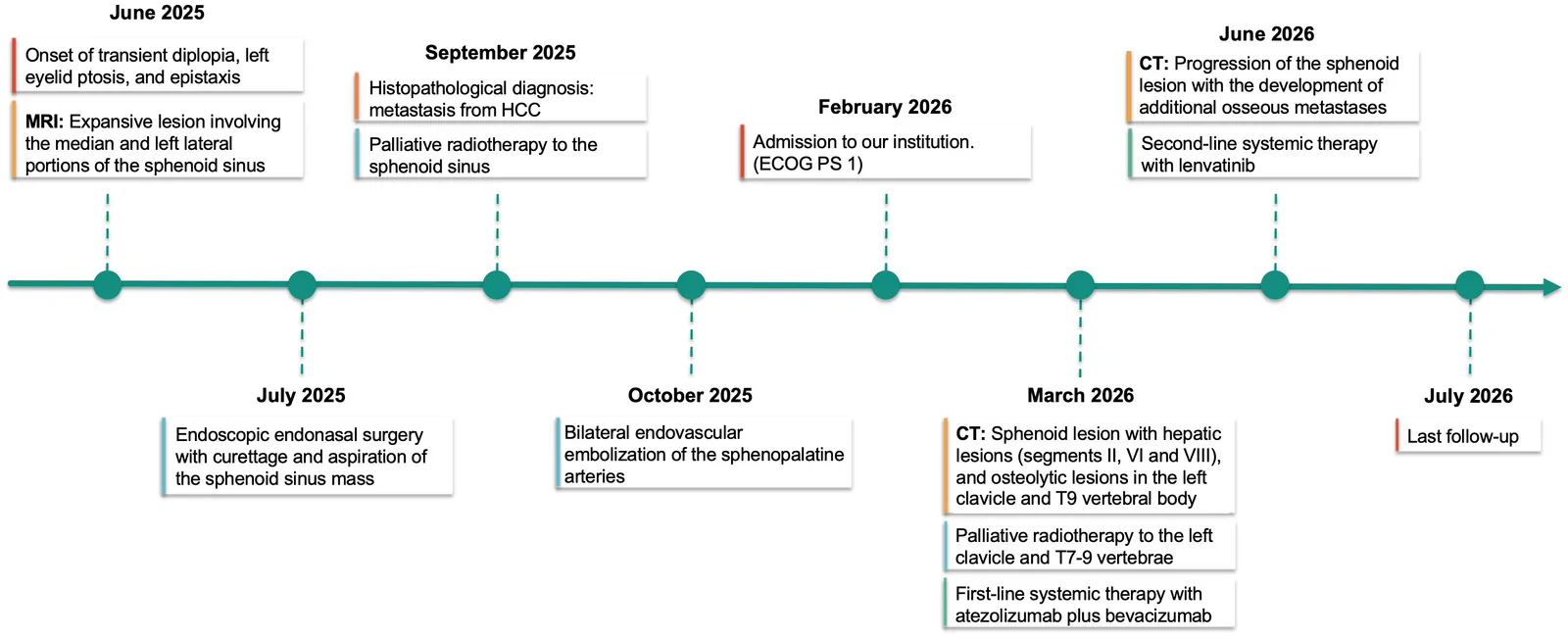
Timeline of clinical events. MRI, magnetic resonance imaging; HCC, hepatocellular carcinoma; ECOG PS, Eastern Cooperative Oncology Group Performance Status; CT, computed tomography.

When extrahepatic spread occurs, the most common sites of metastasis are the lungs and regional lymph nodes, followed by the skeletal system, adrenal glands, and peritoneum (3). Although HCC can disseminate to atypical locations in rare instances, skull base metastases are exceedingly uncommon, with an incidence of 0.4–1.6% (4). Among sinonasal metastatic sites, the maxillary sinus is most frequently involved, whereas sphenoid sinus involvement is uncommon and has mostly been described in isolated case reports (5). We report the case of a patient presenting with neuro-ophthalmic symptoms secondary to sphenoid sinus metastasis, as the first manifestation of a previously undetected HCC. A brief timeline of the patient’s clinical history is reported in **Figure 1**. We present the following case in accordance with the CARE reporting guidelines.

## Case presentation

In June 2025, a 67-year-old man presented to the neurosurgery department of another hospital with complaints of transient episodes of diplopia, left eyelid ptosis, and epistaxis. His medical history was significant only for hepatitis C virus (HCV)-related chronic liver disease, previously treated with antiviral therapy. He had no personal or family history of malignancy, no history of tobacco or alcohol use, and no metabolic risk factors. Despite enrollment in an HCC surveillance program after achieving a sustained virological response, the patient reported poor adherence to scheduled follow-up visits.

Neurological examination revealed left cranial nerves III and IV palsy with associated eyelid ptosis, impaired ocular motility, and loss of pupillary light reflex. In contrast, the right cranial nerves III and IV showed partial involvement, manifesting solely as impaired ocular motility. Ophthalmological evaluation showed a visual acuity of 9/10 in the right eye and 8/10 in the left eye, with normal fundus oculi and intraocular pressure.

**Figure 2:**
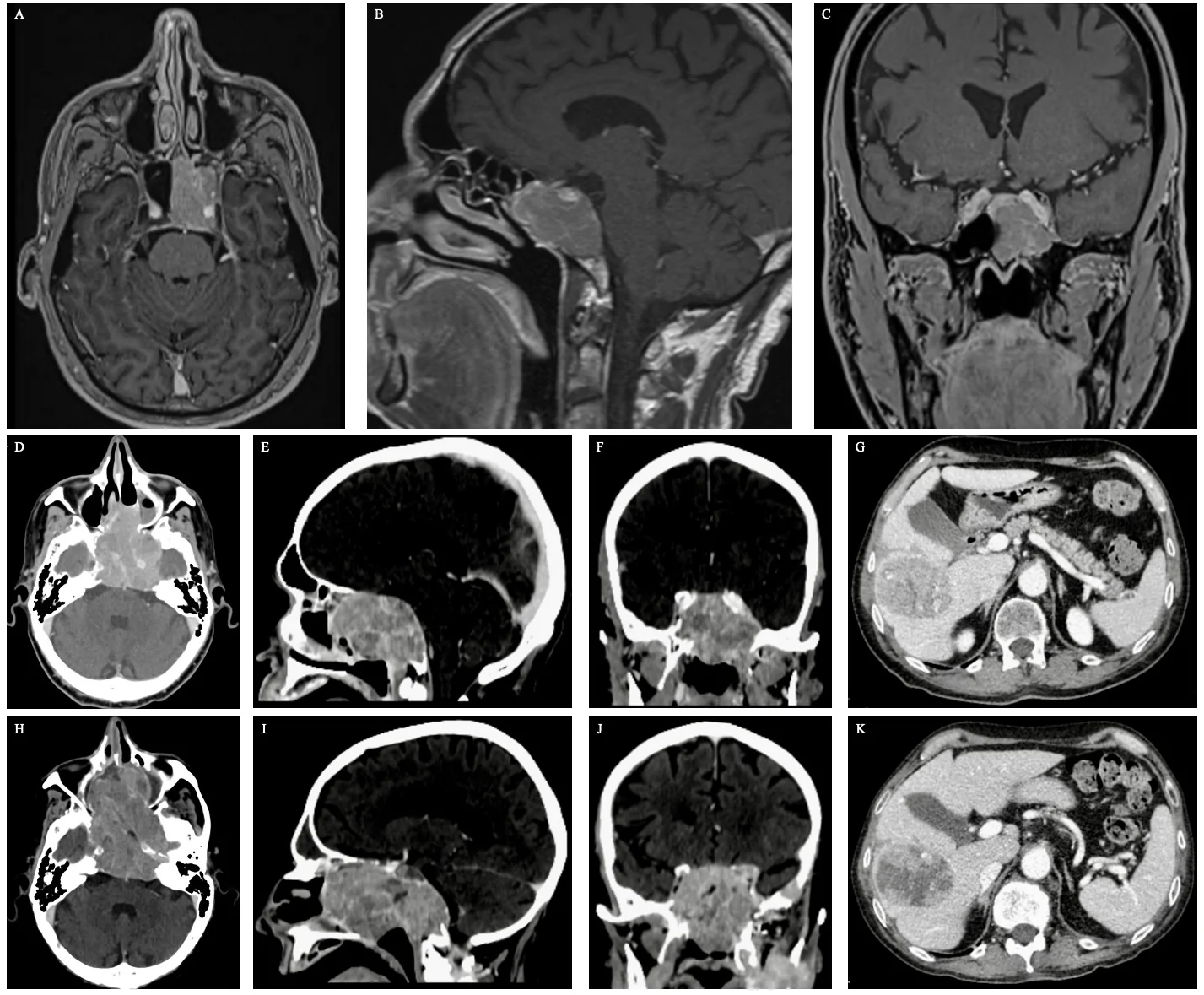
**(A–C)** Contrast-enhanced magnetic resonance imaging (MRI) of the maxillofacial region, obtained in June 2025, in axial **(A)**, sagittal **(B)**, and coronal **(C)** planes demonstrates an expansive mass in the median and left lateral portions of the sphenoid sinus. The lesion infiltrated the upper clivus (maximum dimensions: 42 × 22 mm) and the left cavernous sinus. **(D–G)** Contrast-enhanced computed tomography (CT) scans obtained in March 2026. Craniofacial CT images in axial **(D)**, sagittal **(E)**, and coronal **(F)** planes demonstrate a lesion in the sphenoid region (maximum axial dimensions: 81 × 55 mm). An axial abdominal CT image **(G)** demonstrates a lesion in hepatic segment VI measuring 78 × 59 mm. **(H–K)** Follow-up contrast-enhanced CT scans obtained in June 2026, after four treatment cycles of atezolizumab plus bevacizumab. Craniofacial CT images in axial **(H)**, sagittal **(I)**, and coronal **(J)** planes demonstrate progression of the sphenoid lesion (maximum axial dimensions: 86 × 60 mm). An axial abdominal CT image **(K)** demonstrates the lesion in hepatic segment VI, measuring 76 × 61 mm in maximum axial dimensions.

Contrast-enhanced maxillofacial magnetic resonance imaging (MRI) revealed an expansive mass in the median and left lateral portions of the sphenoid sinus. The lesion infiltrated the upper clivus (maximum dimensions, 42 × 22 mm) and infiltrated the left cavernous sinus (**Figure 2**).

Despite the patient’s known history of HCV-related chronic liver disease, no dedicated preoperative systemic work-up, including serum tumor marker assessment or liver imaging, was performed at that stage.

In July 2025, the patient underwent endoscopic endonasal surgery with curettage and aspiration of the sphenoid sinus lesion for histopathological diagnosis and partial tumor debulking.

**Figure 3:**
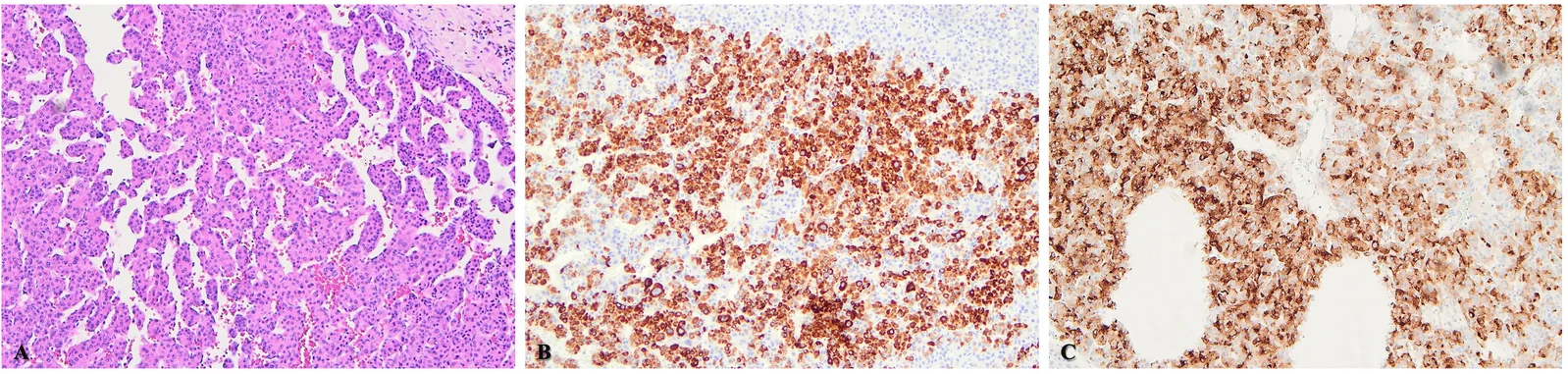
Histopathological **(A)** and immunohistochemical features **(B, C)** of the sphenoid sinus lesion (magnification ×20). **(A)** Hematoxylin and eosin staining showing respiratory mucosa infiltrated by a neoplasm with a solid-to-trabecular architecture. The tumor cells display moderate pleomorphism, round-to-oval nuclei with prominent nucleoli, eosinophilic cytoplasm, and nuclear inclusions. **(B, C)** Immunohistochemical staining showing positivity for hepatocyte antigen **(B)** and glypican-3 **(C)**.

In September 2025, histopathological examination of the surgical specimen revealed a neoplasm with a mixed solid and trabecular architecture. Immunohistochemistry showed diffuse and intense positivity for cytokeratins AE1/AE3 and 8/18, focal reactivity for cytokeratin 20, and multifocal positivity for hepatocyte antigen (HepPar-1) and glypican-3, which was compatible with a diagnosis of HCC (**Figure 3**).

Postoperative contrast-enhanced computed tomography (CT) demonstrated persistent enhancing soft tissue within the sphenoid sinus, previously identified on preoperative MRI, and revealed a hypervascular mass in hepatic segment VI showing washout and a pseudocapsule on delayed-phase images.

**Figure 4:**
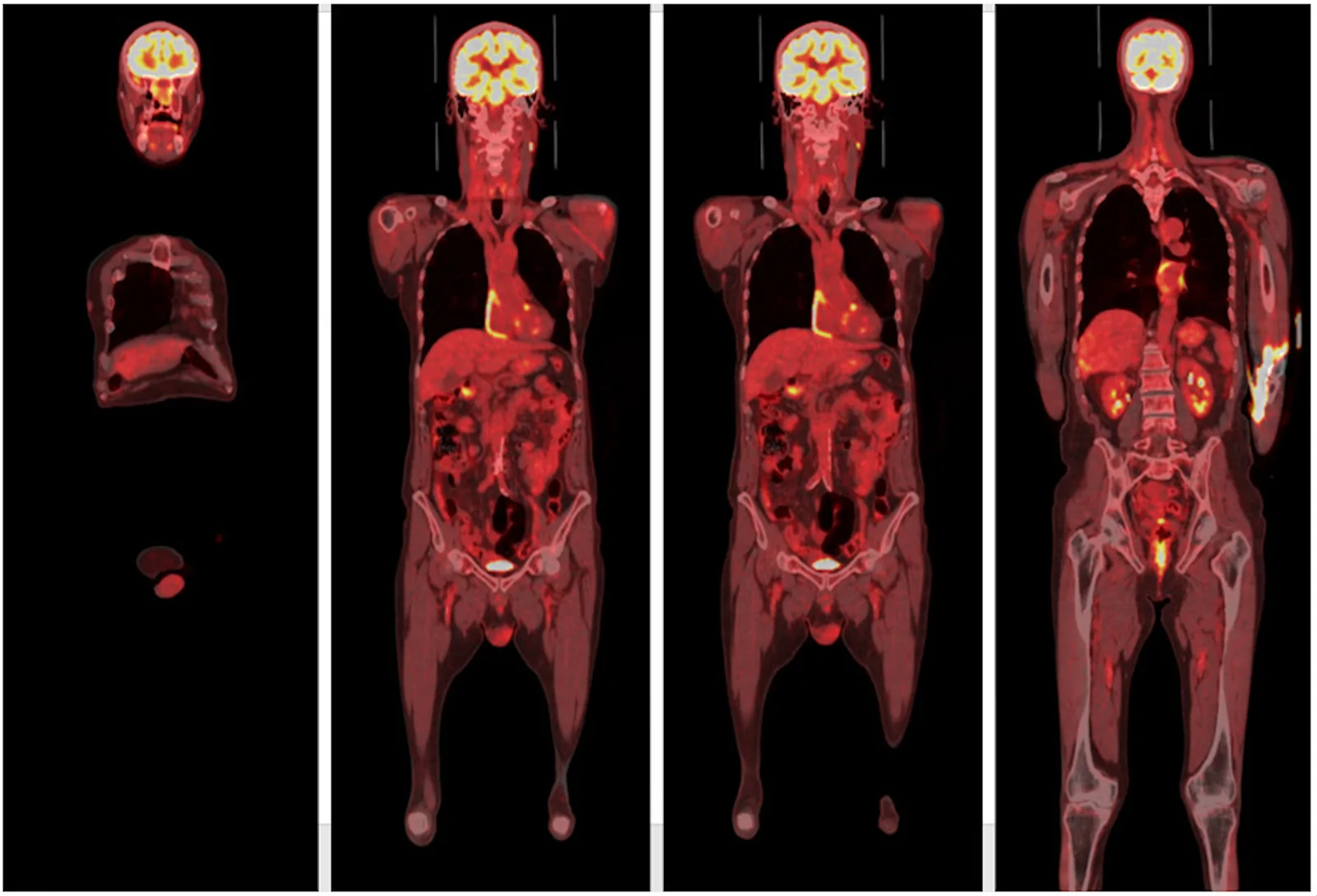
Coronal fusion images of 18F-fluorodeoxyglucose positron emission tomography/computed tomography (18F-FDG PET/CT) scan showing increased 18F-FDG uptake in the sphenoid lesion with ethmoidal extension, in a left retromandibular lymph node, in hepatic segments VI and VII, and nonspecific uptake at the hepatic flexure of the colon.

A subsequent 18F-fluorodeoxyglucose positron emission tomography/computed tomography (18F-FDG PET/CT) scan showed increased uptake in the sphenoid lesion (maximum standardized uptake value [SUVmax] 3.8) with ethmoidal extension (SUVmax 6.6), in a lymph node in the left retromandibular region (SUVmax 8.0), in the VI-VII hepatic segments (SUVmax 3.6), and a nonspecific uptake in the hepatic flexure of the colon (SUVmax 4.6) (**Figure 4**).

In September 2025, to manage the severe pain reported in the left orbital region, the patient underwent palliative radiation therapy to the sphenoid sinus, receiving a total dose of 37.5 Gray (Gy) in 15 fractions. Furthermore, following surgery, the patient experienced recurrent episodes of epistaxis over the subsequent 3 months. Despite conservative pharmacological management, the repeated bleeding resulted in secondary anemia requiring transfusion of packed red blood cells. Consequently, in October 2025, the patient underwent bilateral endovascular embolization of the sphenopalatine arteries via coiling, which successfully achieved stable hemostasis.

Subsequently, the patient chose to continue his oncological management at our center, where he presented in February 2026. At admission, the patient had an Eastern Cooperative Oncology Group (ECOG) performance status of 1. Laboratory evaluation revealed an elevated serum alpha-fetoprotein level (AFP) of 590 ng/mL (reference range: < 7 ng/mL). Liver function was well preserved and classified as Child-Pugh class A. Specific laboratory findings included aspartate aminotransferase (AST) of 60 U/L (reference range: < 50 U/L), alanine aminotransferase (ALT) of 89 U/L (reference range: < 50 U/L), serum albumin of 4.3 g/dL (reference range: 3.5–5.2 g/dL), an International Normalized Ratio (INR) of 1.42 (reference range: 0.80–1.20), and total bilirubin of 0.54 mg/dL (reference range: < 1.20 mg/dL). Similarly, renal function was adequate, with serum creatinine of 1.01 mg/dL (reference range: 0.67–1.17 mg/dL) and sodium of 138 mEq/L (reference range: 135–145 mEq/L). Complete blood count results were within normal limits. Viral serology was negative for hepatitis B surface antigen (HBsAg), and human immunodeficiency virus (HIV) antibodies, while HCV-RNA was undetectable. Neither clinical nor radiological features revealed signs of liver cirrhosis or portal hypertension; specifically, upper gastrointestinal endoscopy confirmed the absence of esophageal and gastric varices.

In March 2026, a new contrast-enhanced CT scan was performed. Imaging revealed the presence of the neoplastic mass in the sphenoid region measuring 81 × 55 mm in maximum axial dimensions. The liver was of normal morphology and volume but contained lesions in segments II (13 mm), VIII (6 mm), and VI (78 × 59 mm), characterized by arterial phase hyperenhancement and delayed washout (**Figure 2**). No splenomegaly, ascites, or collateral venous circulation were identified. In addition, imaging demonstrated new osteolytic lesions involving the proximal third of the left clavicle and the T9 vertebral body with extension to the transverse process.

Disease progression in the sphenoid region, despite prior radiotherapy, and the development of new bone lesions constituted the indication for systemic therapy.

The patient reported that being informed of his cancer diagnosis caused considerable anxiety. The initial presentation, characterized by diplopia, ptosis, and epistaxis, resulted in substantial physical and psychological distress attributable to both the symptom burden and prognostic uncertainty. However, he reported that the previous radiotherapy followed by endovascular embolization markedly improved his quality of life, enabling him to resume his normal daily activities. Despite being fully aware of the advanced stage of his disease, the patient maintained a trusting relationship with the medical team and remained strongly motivated to continue the planned treatment course.

Therefore, in March 2026, the patient underwent additional palliative radiation therapy to the left clavicle and T7–9 with a total dose of 20 Gy in four fractions. During the same month, the patient initiated first-line systemic therapy with atezolizumab plus bevacizumab, completing four treatment cycles by June 2026. Treatment was well tolerated, with no treatment-related adverse events, and was associated with an improvement in the patient’s overall clinical condition. Despite this, in June 2026, the patient developed lumbar spine pain. A contrast-enhanced CT scan demonstrated stable hepatic lesions, alongside an enlargement of the sphenoid lesion (maximum axial dimensions of 86 × 60 mm). This mass extended into and infiltrated the ethmoid air cells, left maxillary sinus, clivus, and left cavernous sinus, with encasement of the intracranial segments of the internal carotid arteries (**Figure 2**). Imaging also revealed the progression of multifocal osseous metastases, including spinal canal involvement at the T9–T10 level, multiple ribs, both scapulae, and the left clavicle. Laboratory investigations showed a marked elevation in serum alpha-fetoprotein (AFP: 3234.0 ng/mL). Liver function tests indicated moderate cytolysis (AST: 107 U/L, ALT: 209 U/L) with preserved synthetic function (serum albumin: 3.8 g/dL, INR: 1.18) and normal total bilirubin (0.39 mg/dL), while the complete blood count showed no clinically significant abnormalities.

In light of disease progression according to modified Response Evaluation Criteria in Solid Tumors (mRECIST), second-line targeted therapy with lenvatinib, at a dose of 12 mg once daily, was initiated in June 2026. The patient maintained a positive attitude and agreed to proceed with the proposed treatment. At the most recent follow-up in July 2026, the patient had completed one cycle of lenvatinib without adverse events and therapy was ongoing.

Ethical approval is not required for this study, in accordance with local or national guidelines. Written informed consent for the publication of this case report, including radiographic images and patient pictures, was obtained from the patient. A copy of the written consent is available for review by the journal’s editorial office.

## Discussion

Metastatic spread to the paranasal sinuses is unusual in cancer patients, and typically occurs in advanced disease. The most frequently reported primary malignancies spreading to this region are renal, lung, and breast cancers, followed by urogenital and gastrointestinal tract tumors (6).

Among the sinonasal cavities, the maxillary sinus is the most commonly involved; conversely, secondary localization in the ethmoid, frontal, and sphenoid sinuses or the nasal cavity is far less frequent (7).

While extrahepatic spread of HCC occurs in approximately 30–50% of patients, primarily targeting the lungs (43.8–77.8%), bones (16–52.5%), and lymph nodes (17.5–26%), metastases to the head and neck remain exceedingly rare (3). In 2007, Huang et al. (8) documented only 103 cases. Although a few subsequent cases have been reported in the literature, sphenoid sinus involvement remains exceedingly rare. Metastases to the sphenoid sinus present with a broad and nonspecific range of clinical signs and symptoms, reflecting the diverse anatomical structures located within this region. Clinical manifestations may include retro-orbital and facial pain, epistaxis, nasal swelling, nasal obstruction, facial masses and headaches. Furthermore, neurological and ophthalmological findings, such as ophthalmoplegia, diminished corneal reflex, hyperesthesia, dysesthesia, ptosis, diplopia, eyelid edema, epiphora, and proptosis, are potential clinical presentations (6–9).

Sphenoid sinus lesions may result from a wide spectrum of primary benign and malignant conditions, as well as metastatic disease. The differential diagnosis includes inflammatory disorders, such as fungal ball (mycetoma), mucocele, and osteomyelitis; benign primary sinonasal tumors, including hemangioma, ossifying fibroma, pituitary adenoma, and sinonasal papilloma; and primary malignant sinonasal tumors, including chordoma, nasopharyngeal carcinoma, neuroendocrine tumors, lymphoma, and plasmacytoma or multiple myeloma (10).

Given the lack of pathognomonic symptoms to differentiate metastatic lesions from other diseases involving the sphenoid sinus, diagnostic imaging and biopsy are mandatory to confirm the malignant nature of the lesion, characterize its histological features, and enable immunohistochemical analyses to identify the primary site of origin. The exact mechanism by which HCC metastasizes to the head and neck region remains unclear. Two main pathways have been proposed: hematogenous dissemination and lymphatic spread of the disease. The first mechanism involves hematogenous spread via the systemic circulation. Tumor emboli enter the caval venous system, pass through the right heart, reach the pulmonary circulation, and subsequently disseminate through the arterial system to the head and neck region (11). However, this pathway usually presupposes the presence of synchronous or metachronous pulmonary metastases. In cases such as ours, where sinonasal metastasis occurs without pulmonary involvement, an alternative mechanism may involve Batson’s vertebral venous plexus. This valveless system extends from the skull base to the coccyx and connects the venous systems of the thorax, abdomen, and pelvis. Under conditions of marked increase in intra-abdominal or intra-thoracic pressure, retrograde blood flow can divert tumor cells from the caval system into the epidural plexus, allowing them to bypass the pulmonary circulation and directly reach cranial structures with rich venous interconnections, thereby facilitating direct metastasis to the paranasal sinuses. Lymphatic spread is an alternative route of dissemination. In this scenario, tumor emboli from regional lymph nodes may flow into the thoracic duct and then migrate retrogradely toward the head and neck region via the intercostal, mediastinal, or supraclavicular lymphatic vessels (7).

Over the last decades, many advances have been made in the management of advanced HCC (**Table 1**). A paradigm shift occurred with the introduction of novel combinations involving immune checkpoint inhibitors with anti-VEGF or anti-PD-1 plus anti-CTLA-4 into the clinical landscape. Indeed, as evidenced by the CheckMate 9DW and Himalaya trials, the combination of nivolumab plus ipilimumab or durvalumab plus tremelimumab has proven to be more effective than sorafenib or lenvatinib monotherapy (12, 13). Simultaneously, the combination of atezolizumab and bevacizumab has shown significantly improved overall survival, progression-free survival, and overall response rate compared to sorafenib (14). Despite these therapeutic advances, the prognosis for patients with paranasal sinus metastasis remains poor (7), often reflecting a high systemic tumor burden or aggressive biological behavior of the tumor.

**Table 1:** Summary of phase III trials of approved first-line treatment in advanced hepatocellular carcinoma.

Trial/Parameter	Sharp (17)	Reflect (18)	IMbrave150 (14)	Himalaya (13)	Checkmate 9DW (12)
Primary Endpoint	OS and Time to symptomatic progression	OS	OS and PFS	OS	OS
Experimental Arm	Sorafenib vs. Placebo	Lenvatinib vs. Sorafenib	Atezolizumab + Bevacizumab vs. Sorafenib	-Tremelimumab + Durvalumab vs. Sorafenib -Durvalumab vs. Sorafenib	Nivolumab-Ipilimumab vs. Sorafenib/Lenvatinib
OS (mo) HR	10.7 vs. 7.9; HR: 0.69	13.6 vs. 12.3; HR: 0·92	19.2 vs 13.4; HR: 0.66	-16.4 vs 13.7; HR: 0.78 -16.6 vs 13.7; HR: 0.86	23.7 vs 20.6; HR: 0.79
PFS (mo) HR	5.5 vs. 2.8; HR: 0.58	7.4 vs. 3.7; HR: 0.66	6.9 vs 4.3; HR: 0.65	-3.8 vs 4.0; HR: 0.90 -3.6 vs 4.0; HR: 1.02	9.1 vs 9.2; HR: 0.87
ORR %	2% vs 1%	24% vs 9%	30% vs 11%	-20% vs 5% -17% vs 5%	36% vs 13%

PFS, progression-free survival; OS, overall survival; ORR, objective response rate; HR, hazard ratio; mo, months.

Currently, there are no established guidelines that provide clear recommendations for the optimal management of these patients.

Therefore, a multidisciplinary team discussion is mandatory to individualize treatment planning for this rare metastatic presentation. Local modalities, such as surgical and/or radiotherapeutic approaches, may be considered to reduce the tumor burden and alleviate compressive symptoms exerted by the mass on adjacent structures (7). Radiotherapy can facilitate the shrinkage of paranasal metastases, induce fibrosis in the surrounding osseous structures, and control epistaxis (15). Conversely, surgical intervention is not routinely indicated in patients with metastatic disease. It should be strictly reserved for diagnostic purposes, local tumor debulking, or cases in which radiotherapy is contraindicated or not feasible. In advanced disease, surgery can inadvertently delay the initiation of systemic therapy. Furthermore, the infiltrative nature of HCC metastasis within the sphenoid sinus makes complete surgical resection difficult (9).

In cases of refractory epistaxis, angiographic embolization of the feeding vessels is an additional viable palliative procedure (16). Regarding systemic management, no data are available to guide the selection of an optimal therapeutic regimen for these patients. Moreover, no clinical trials have demonstrated a clear clinical benefit associated with one specific combination therapy over another among currently available options. Consequently, therapeutic decision-making must be primarily based on the patient’s comorbidities, overall clinical condition, disease burden, related symptomatology, and the potential of a given regimen to elicit a rapid tumor response.

In summary, this case, characterized by an atypical initial manifestation of a previously occult primary HCC, highlights the aggressive biological behavior of HCC and its propensity to involve rare metastatic sites, such as the paranasal sinuses. This case emphasizes the need for clinicians to carefully evaluate any anomalous masses in patients with chronic liver disease, as they may represent metastatic spread from an undetected primary malignancy.

Nevertheless, several limitations should be acknowledged. As a single-case report, its findings cannot be generalized. Moreover, the relatively short follow-up precludes definitive conclusions regarding the long-term efficacy of systemic therapy, while the prolonged interval between the establishment of the diagnosis and the initiation of treatment for the underlying disease may have influenced the clinical course. In addition, only limited clinical documentation was available from the referring hospital, restricting the completeness of the reconstruction of the patient’s initial diagnostic and therapeutic pathway. Furthermore, the proposed mechanisms underlying metastatic dissemination remain hypothetical and require further investigation.

## Data Availability

The raw data supporting the conclusions of this article will be made available by the authors, without undue reservation.
